# Single and co-inoculum of endophytic bacteria promote growth and yield of Jerusalem artichoke through upregulation of plant genes under drought stress

**DOI:** 10.1371/journal.pone.0286625

**Published:** 2023-06-02

**Authors:** Patcha Boonmahome, Junthima Namwongsa, Nimitr Vorasoot, Sanun Jogloy, Nuntavan Riddech, Sophon Boonlue, Wiyada Mongkolthanaruk

**Affiliations:** 1 Faculty of Science, Department of Microbiology, Khon Kaen University, Khon Kaen, Thailand; 2 Faculty of Agriculture, Department of Plant Science and Agricultural Resources, Khon Kaen University, Khon Kaen, Thailand; Universidade de Coimbra, PORTUGAL

## Abstract

*Helianthus tuberosus* L. (Jerusalem artichoke) produce inulin, a type of fructan, which possesses several biotechnology applications, e.g., sugar syrup, prebiotics, fiber in diabetic food, enabling blood sugar and cholesterol reduction. Drought reduces inulin accumulation in the tubers of Jerusalem artichoke as the plants protect themselves from this stress by induction of stress gene responses, effecting growth reduction. Endophytic bacteria are promising candidates to promote plant growth and yield particularly under abiotic stress. Therefore, three endophytic bacteria with plant growth promoting properties were examined for their ability to improve Jerusalem artichoke growth and yield under both well-watered and drought conditions when inoculated individually or in combinations in pot experiments with 2 factorial random complete block design. The interactions of the endophytic bacteria and plant host determined the differential gene expression in response to drought as revealed by quantitative polymerase chain reaction. Single inoculum of the endophytic bacteria increased the height, weight, root traits, and harvest index of Jerusalem artichoke compared to co-inocula under both well-watered and drought conditions. However, the co-inocula of *Rossellomorea aquimaris* strain 3.13 and *Bacillus velezensis* strain 5.18 proved to be a synergistic combination leading to high inulin accumulation; while the co-inocula of *B*. *velezensis* strain 5.18 and *Micrococcus luteus* strain 4.43 were not beneficial when used in combination. The genes, dehydrin like protein and ethylene responsive element binding factor, were upregulated in the plants inoculated with single inoculum and co-inocula of all endophytic bacteria during drought stress. Moreover, the gene expression of indole-3-acetic acid (IAA) amido synthetase were up-regulated in Jerusalem artichoke inoculated with *M*. *luteus* strain 4.43 during drought stress. The fructan:fructan 1-fructosyltransferase (1-FFT) was also stimulated by the endophytic bacteria particularly in drought condition; the results of this study could explain the relationship between endophytic bacteria and plant host for growth and yield promotion under well-watered and drought conditions.

## Introduction

An inulin-type fructan is fructo-polysaccharide with linear β-(1,2) linkages with a terminal glucose residue at a low degree of polymerization (DP). Inulin can be utilized as fructose syrup (sweetening agent) in drink products, and as a prebiotic or fat replacement in food products [1]. Jerusalem artichoke (*Helianthus tuberosus* L.) is an inulin (fructan) accumulating plant which synthesizes inulin by the reaction of sucrose:sucrose 1-fructosyltransferase (1-SST; EC 2.4.1.99) and fructan:fructan 1-fructosyltransferase (1-FFT; EC 2.4.1.100) using sucrose as a substrate. The 1-SST enzyme catalyzes the biosynthesis of the trisaccharide 1-kestotriose firstly, and consequently the 1-FFT catalyzes the elongation of 1-kestotriose to inulin. The 1-FFTs prefer 1-kestotriose or very short inulin molecules as acceptor substrate [2]. Another enzyme, fructan 1-exohydrolase (FEHs; EC 3.2.1.153) hydrolyzes inulin into free sugars (sucrose, glucose, and fructose) in response to cold temperatures [3].

Drought affects tuber yield, total biomass, harvest index and the water use efficiency of Jerusalem artichoke. Tuber and biomass reduce by 85–98% under severe drought and the reduction of these parameters stand at 49–85% in moderate drought [4]. Jerusalem artichoke of different varieties behave differently in their photosynthetic and physiological responses to drought. Some varieties with high tuber yield are drought susceptible while some with low tuber yield are highly tolerant to drought. The drought tolerant genotypes exhibit high tuber production in water deficit as they retain leaf area, photosynthesis rate, and stomatal conductance [5]. Moreover, plants increase stress tolerance involved in various molecular networks including signal molecules. The action of phytohormones and complex regulator, e.g., abscisic acid (ABA), indole-3-acetic acid (IAA) signals [6], ion transport, and the activities of transcription factors, is key to plant responses to adapt and survive. Amino acids are common factors responding to drought stress in plants; likely, Jerusalem artichoke accumulates phenylalanine in the leaves as an adaptation to drought stress [7]. Moreover, transcription factors regulate gene expression in metabolic pathways may respond to drought stress. The transcription factors of NAC, MYB, WRKY, homeobox-leucine zipper (HD-ZIP) and basic leucine-zipper (bZIP) were analyzed for their role in the drought resistance mechanism of Jerusalem artichoke; these genes related to the response to polyethylene glycol-simulated drought stress [8].

Dehydrins are proteins that express and accumulate in plants under abiotic stress including drought, heat, frost, metals/metalloids, or salinity. They stabilize biomolecules and membranes during dehydration stress and may have a role of chaperone functions [9]. Ethylene responsive element binding factors (ERFs) are transcription factor proteins that are specific to ethylene response genes for pathogens and environmental changes [10]. HD-Zip genes act by regulating plant architecture, organogenesis, and reproductive processes. Most of the HD-Zip genes are involved in the mediation of external signals to regulate plant growth [11]. The role of HD-Zip I subfamily genes is to maintain plant growth under water deficit conditions. Indole-3-acetic acid amido synthetase (GH3) catalyze amino acid conjugation into auxin hormones (IAA, jasmonic acid, salicylic acid); auxin signaling is involved in plant growth, development and defense systems [12]. These genes are a major complex response to drought and are involved in IAA which relates to induced systemic tolerance (IST) under osmotic stress [13]; thus, these genes were selected to represent an overview of plant and endophytic bacteria interaction under drought stress in this study.

Endophytic bacteria have roles of phosphate solubilizing, nitrogen fixation, auxin production, 1-aminocyclopropane-1-carboxylate (ACC) deaminase for plant growth promotion and protection from environmental stresses. They colonize within various plant parts and have direct relation with plants and are incorporated to phytobiome for new functional traits [1,14], compared to the rhizospheric bacterial community which colonize through root tip, leading to root hairs and lateral roots [15]. The rhizobacteria present many beneficial properties especially nutrient supply and heavy metal decontamination in the soil [16]. Reduced yield and biomass of Jerusalem artichoke could be revered using microbial inocula under drought stress. The arbuscular mycorrhizal fungi which colonize plant root improve drought tolerance in Jerusalem artichoke by the mechanism of phosphate solubilization and transportation to plants with the synergistic action of phosphate solubilizing bacteria [17]. The endophytic bacteria also possess properties to promote growth and yield of Jerusalem artichoke [18,19]. Additionally, the organic fertilizer helps by improving the water status of Jerusalem artichoke exposed to drought stress; the organic materials, such as rice straw compost and humic acid, improve soils physical condition and water holding capacity, subsequently boosting the supply of plant nutrients [20].

Endophytic bacteria, which possess properties of IAA, inulinase (inulin degradation) and levansucrase (inulin synthesis) production, were chosen from previous study to investigate their actions between single and co-inoculation for promoting the growth and yield (inulin) of Jerusalem artichoke under well-watered and drought conditions. These endophytic bacteria with different characteristics might perform a synergistic action to enhance drought tolerance in HEL65 variety, which was the drought moderate genotype with low tuber yield and low drought tolerance. Moreover, plant genes involved in inulin synthesis (1-SST, 1-FFT, 1-FEH) exhibited a relationship between the endophytic bacteria and plant host for inulin promotion. The genes response to drought in plants were determined to gauge the stimulation of the endophytic bacteria involved in plant gene responses.

## Materials and methods

### Endophytic bacteria inoculum

Endophytic bacteria isolated from the leaf and stem of Jerusalem artichoke in the previous study were *Bacillus aquimaris* strain 3.13 (MH973229), which was transferred to a new genus as *Rossellomorea aquimaris*, *Micrococcus luteus* strain 4.43 (MH973230) [19] and *Bacillus velezensis* strain 5.18 (MH973231) [21]. The bacteria were grown in nutrient broth (NB) and incubated at 30°C with agitation of 150 rpm for 24 hours. The cell suspension was centrifuged at 2,800 g, 15 min to collect the cell pellet which was washed twice with 0.85% NaCl. The cell was resuspended in sterile distilled water and adjusted equal to McFarland standard No. 0.5 which compared to cell density at approximately 1 x 10^8^ CFU/ml. The cell suspension was used as inoculum.

### Effects of endophytic bacteria on plant growth and yield under normal and drought conditions

#### Soil preparation

Soil collected from local agricultural land was characterized as sandy loam soil containing 0.08% nitrogen (N) using micro-Kjeldahl method [22], 80 mg kg^-1^ total phosphorus (P) using molybdenum blue method [23], 797.46 mg kg^-1^ total potassium (P) using wet oxidation method [23], available P by Bray II–molybdenum blue method [24], K and Ca using flame photometer [25] at 2.57, 43.85 and 260 mg kg^-1^, respectively. The soil contained organic matter of 0.44%, determined by Walkley and Black method [22], while analysis with a conductivity meter revealed electrical conductivity at 0.02 dS m^-1^. The unsterilized soil was passed through a 2 mm sieve and then packed into a pot (15-inch diameter, 11-inch depth) divided into 2 layers; 16.5 kg of soil was put at the bottom of the pot and the top of the pot was filled with 6.5 kg of soil. A drip line was installed in the pot between the 2 layers of soil and connected with plastic tubes to supply water from the surface soil. Water was supplied at field capacity level before 1 day of planting.

#### Experimental design for planting

Jerusalem artichoke (HEL65) received from Plant Science and Agricultural Resources, Faculty of Agriculture, Khon Kaen University was prepared by cutting tubers into small pieces with 4–5 buds each. The tuber pieces were incubated in burnt rice husk in plastic boxes for 7 days and well-watered to induce germination. The germinated plantlets were then transferred into a mixture of soil and burnt rice husk (1:1) in plastic trays and well-watered until the plantlets had 2–3 leaves (about 7 days), after which the plantlets were transplanted into the pot with field capacity of watering for 7 days before inoculating with bacteria. The pot experiments were set up at Agronomy farm, Khon Kaen University (16.4740°N, 102.8220°E), Khon Kaen in June-October, 2019. Air temperatures averaged between 20.1–33.1°C; the average solar radiation was 14.7 MJ m^-2^ d^-1^, and relative humidity values averaged 89.5%. The experiments were arranged in a two-factorial experiment with randomized complete block design (RCBD) with replication in 4 blocks (total number of 128 pots). Treatments in this experiment are shown in Table 1. A single inoculum (5 ml) of each strain was inoculated into the seedling in each pot; 2.5 ml of each strain was transferred into each pot for co-inoculation; 1.67 ml of each strain was transferred into each pot for triple inoculation; and 5 ml of sterile distilled water was inoculated into the pot as a control. The inoculums were added to each pot once again after 30 days of planting to maintain bacterial cell number of the endophytic bacteria. During planting, no fertilizer was applied; weeds and pests were controlled manually using local plant (*Azadirachta indica* A. Juss) extracts.

**Table 1:** Treatments used to evaluate plant growth under normal and drought conditions by controlling soil moisture content in pot experiments.

Treatment of planting	Description
T1	Inoculated *R*. *aquimaris* 3.13
T2	Inoculated *M*. *luteus* 4.43
T3	Inoculated *B*. *velezensis* 5.18
T4	Co-inoculated *R*. *aquimaris* 3.13+*M*. *luteus* 4.43
T5	Co-inoculated *R*. *aquimaris* 3.13+*B*. *velezensis* 5.18
T6	Co-inoculated *M*. *luteus* 4.43+*B*. *velezensis* 5.18
T7	Co-inoculated *R*. *aquimaris* 3.13+*M*. *luteus* 4.43+*B*. *velezensis* 5.18
T8	Non-inoculated control
Treatment of watering	Soil moisture content (%)
W1—Well watering (full available soil water)	
30 DAT	14.42
75 DAT	13.85
125 DAT	13.06
W2—Severe drought (1/3 available soil water)	
30 DAT	14.23
75 DAT	7.80
125 DAT	7.74

DAT mean days after transplanting.

#### Water management

Levels of watering were calculated by following Janket et al. [26]. Where, ETcrop = ETo × Kc which ETo (mm) is evapotranspiration of a reference plant calculated by pan evaporation method; Kc is the crop water requirement coefficient for sunflower at various growth stages as there is no data on the crop coefficient of Jerusalem artichoke. There were 2 levels of watering as follows: W1 referred to well-watering, equivalent to the crop water requirement (ET crop, mm/day). W2 referred to watering 1/3 of the amount supplied under W1. The water was supplied at W1 for 30 days of planting and then the reduction of water supply was applied in W2 until harvesting time, measuring soil moisture content (Table 1) to regulate water supply.

### Analysis of plant parameters

Plants were harvested at 75 and 125 days after transplanting (DAT) for determination of plant parameters. The height of plants was measured from the soil surface to the tips of the top leaves; after that the plants were cut and separated into shoots and roots. The roots were washed with water to remove soil and air dried. Fresh weights of stem, leaves and root were measured on a scale; after that the plant samples were dried in an oven at 70°C until constant dry weights. For root parameters, a random sample of fresh root (10% of each treatment) was taken to scan using root scanner (Epson perfection V700 photo) and data analysis was performed by Win Rhizo program for root length, root diameter, root volume and root surface area. For leaf parameters, a random sample of fresh leaves was measured for leaf area by LI-3100C area meter (LI-COR Bioscience). SPAD chlorophyll was detected from the second expanded leaves from the top of main stem using SPAD meter (Konica monita, Japan). The same leaves from each pot were analysed for photosynthetic rate, stomatal conductance and transpiration rate using a LI-6400XT portable photosynthesis system (LI-COR Bioscience). Water use efficiency (WUE) was calculated from photosynthetic rate divided by transpiration rate. Relative water content (RWC) was determined to evaluate plant water status following the method described by Janket et al. [26].

For yield at harvest time (125 days), the tubers were washed with water, separated from roots and air dried to remove the moisture; after that the tubers of each plant were counted and both fresh and dry weight was recorded. The harvest index was calculated following the formula: Harvest index = total dry weight of tubers/ total dry biomass (shoots, roots and tubers). Finally, the dry tubers were ground into fine powder; 2 g of the powder was added to 25 ml of distilled water, incubated at 80°C for 20 min, centrifuged at 1,792 g for 5 min and filtered through membrane (Whatman No.4). A 500 μl of the filtrates was mixed with 0.75 ml of 3% HCl and the volume adjusted to 25 ml using distilled water. The mixture was boiled at 100°C for 45 min, cooled on ice and the absorbance measured at 390 nm to compare inulin content from a standard curve of fructose [27].

Proline was detected from leaves using 0.5 g in 2 ml of 3% sulfosalicylic acid; the samples were mixed by vortex mixture and filtered through filter membrane (Whatman No. 4). The filtrate was mixed with 2 ml of ninhydrin acid and 2 ml of glacial acetic acid and then incubated at 100°C for 60 min. The mixture was cooled on ice and mixed with 4 ml of toluene. After that the color product appeared in the upper phase, measured at 520 nm calculated with proline standard [28].

### Determination of plant gene expression

#### RNA extraction and cDNA synthesis

Total RNA was extracted from 100 mg of plant tissue, leaves, stems and roots at 75 and 125 DAT. The plant samples were ground in liquid nitrogen with mortar and pestle. The procedure was followed by GeneJET plant RNA Purification Mini Kit (Thermo Scientific) and then RNA was treated with DNaseI (Thermo Scientific). Total RNA was determined using Denovix DS-11 Spectophotometer (DeNovix, USA) at 260 nm and 280 nm for quantity and quality RNA, respectively, and was examined with 1% of agarose gel electrophoresis.

cDNA was performed by RevertAid first stand cDNA synthesis kit (Thermo Scientific) using 0.5 μg of total RNA mixed with 1 μl of oligo (dT)18 primer for the first strand synthesis. The mixture was incubated at 65°C for 5 minutes. After incubation, the second strand cDNA was synthesized in a reaction mixture as follows: 4 μl of 5X Reaction Buffer, 1 μl of RiboLock RNase Inhibitor (20 U/μl), 2 μl of 10 mM dNTPs Mix and 1 μl of RevertAid M-MuLV RT (200 U/μl). The reaction was incubated at 42°C for 60 min followed by heating at 70°C for 5 minutes. The cDNAs were diluted to a final concentration of 200 ng/μl with sterile ultrapure water prior to qRT-PCR analysis.

#### Plant gene analysis by qRT-PCR

The gene expressions were determined by quantitative real time PCR (qRT-PCR) technique in a 96 well plate with LightCycler^®^ 480 instrument (Roche Molecular Biochemicals, USA). Total 10 μl of reaction mixture composed of 1.6 μl of ultrapure water, 5 μl of 10 mM dNTP mix, 3 μl of cDNA template, 0.2 μl of each specific primers (S1 Table) and the mixture of Maxima SYBR Green qPCR Master Mix (2x) and provided ROX Solution (Thermo Scientific). The thermal cycling conditions were followed at 95°C for 3 min for pre-denaturation, and 50 cycles of 95°C for 20s, 55°C for 20s, and 72°C for 30s. The experiments were performed in three replicates for each sample. The data were shown in Ct values and the relative expression levels were calculated as 2-ΔΔCt method (2-(ΔCt of treatment-ΔCt of control)).

### Statistical analysis

The data of plant growth and yield were subjected to two-way analysis of variance (ANOVA) using STATISTIX8 software and homogeneity of variance was performed by all pairwise comparisons with LSD method at 95% and 99% significant differences for all parameters. Pearson’s correlation coefficient between plant parameters was calculated using Excel program, comparing the calculated number and critical r values at probability levels of P < 0.05 and P < 0.01.

## Results

### Bacterial inoculums promoted plant growth under normal and drought conditions

**Fig 1:**
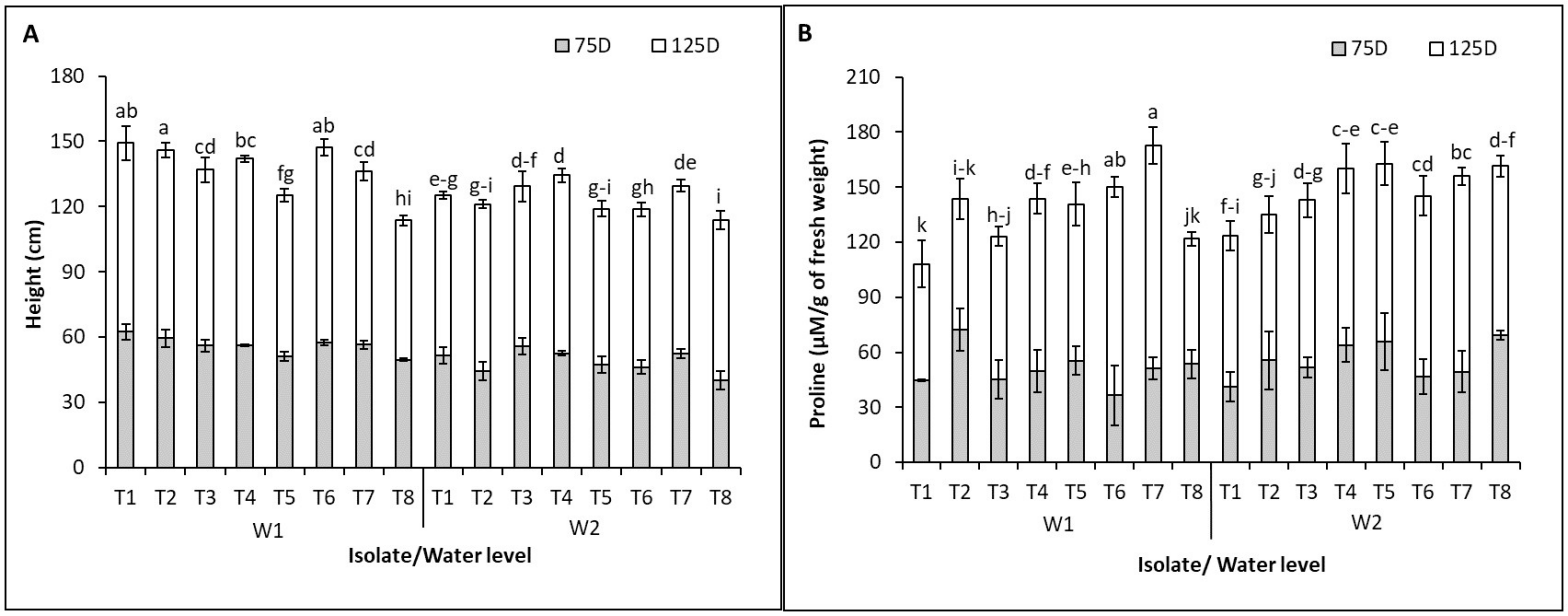
Effect of the endophytic bacteria on plant height (A) and proline accumulation (B) between well watering and drought stress at 75 and 125 days after planting. Bars indicate standard deviation followed by the different letters that are significantly different at P<0.05. W1-W2 represent treatment of watering; T1-T8 represent treatment of planting described in Table 1.

Plant height was significantly higher with bacterial inoculation compared with the control under well-watering; both single or co-inoculations of the bacterial strains showed significantly improved plant height when compared with control (Fig 1A). However, the plants co-inoculated with the *R*. *aquimaris* 3.13+*B*. *velezensis* 5.18 were significantly shorter than other treatments. The co-inocula of *M*. *luteus* 4.43 resulted in height promotion under both conditions either with *R*. *aquimaris* 3.13 or *B*. *velezensis* 5.18 or mixed all strains; in contrast, the co-inocula of *R*. *aquimaris* 3.13+*B*. *velezensis* 5.18 showed negative results under well-watering. Drought stress showed negative effects on Jerusalem artichoke growth with or without the endophytic bacteria, leading to height reduction. *B*. *velezensis* 5.18 induced height promotion similar to the co-inocula of *R*. *aquimaris* 3.13+*M*. *luteus* 4.43 and triple-inocula under drought stress. While *M*. *luteus* 4.43 and the co-inocula of *R*. *aquimaris* 3.13+*B*. *velezensis* 5.18 showed no significant differences when compared with the control. Proline accumulation in the plant was high in the treatments of co-inoculation under well-watering, particularly the co-inocula and the triple-inocula which showed significantly higher proline levels than the control (Fig 1B). The single inoculum of all strains reduced proline accumulation in Jerusalem artichoke under drought condition, implying that the endophytic bacteria reduced plant stress. Conversely, high proline content indicated plant response to stress for adaptation and survival.

### Biomass and yield of Jerusalem artichoke inoculated with endophytic bacteria

**Fig 2:**
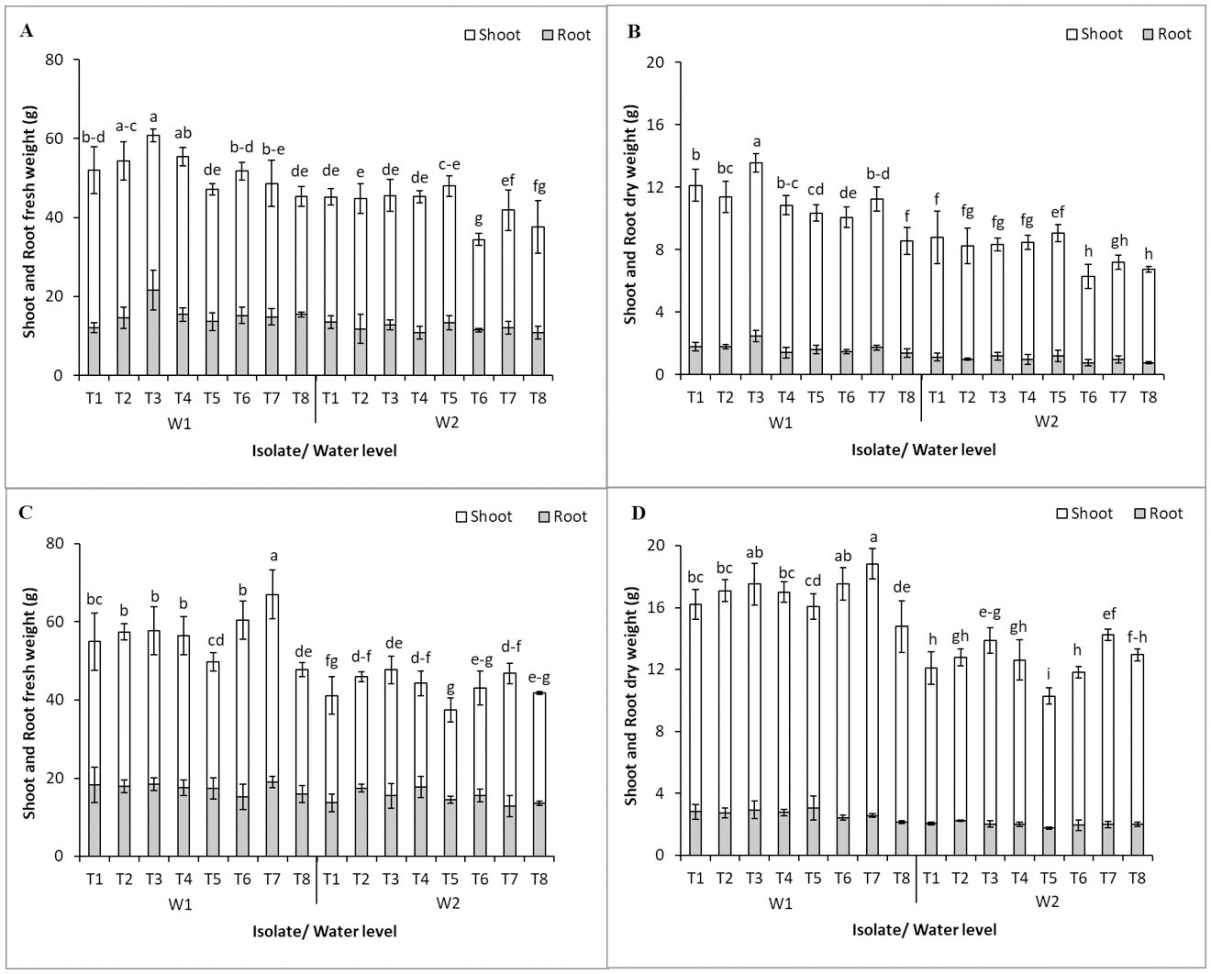
Biomass of Jerusalem artichoke due to planting in normal (W1) and drought condition (W2) inoculated with single inoculum and co-inocula of endophytic bacteria at 75 (A, B) and 125 (C, D) days after planting. T1-T8 represent treatment of planting described in Table 1. Bars indicate standard deviation followed by the different letters that are significantly different at P<0.05.

Under well-watering, the plants inoculated with the bacterial strains recorded higher root and shoot fresh weight and dry weight than the control; the results showed clearly at 125 DAT (Fig 2). *M*. *luteus* 4.43, *B*. *velezensis* 5.18 and co-inocula of *R*. *aquimaris* 3.13+*M*. *luteus* 4.43 promoted shoot and root fresh weight significantly compared with the control at 75 DAT (Fig 2A) and all inoculations promoted shoot and root fresh weight significantly at 125 DAT, except the co-inocula of *R*. *aquimaris* 3.13+ *B*. *velezensis* 5.18 (Fig 2C); these results accorded with the shoot and root dry weight results (Fig 2B and 2D). In drought stress, the shoot and root fresh weight of the plants with single inoculation was significantly higher than the control including the co-inocula of *R*. *aquimaris* 3.13+ *M*. *luteus* 4.43 and that of the *R*. *aquimaris* 3.13+*B*. *velezensis* 5.18 at 75 DAT. Fresh weight did not significantly differ from the control at 125 DAT; the dry weight indicated the same results as the fresh weight under drought condition. However, the coinoculation of *R*. *aquimaris* 3.13+*B*. *velezensis* 5.18 and *M*. *luteus* 4.43+*B*. *velezensis* 5.18 resulted in the lowest shoot and root weight (both fresh and dry weight) under drought condition at 125 DAT; even so, the single inoculum of *B*. *velezensis* 5.18 was the best treatment for promoting biomass. At 75 DAT factorial analysis showed significant effects of each independent factor (water levels and bacterial inoculation) on all plant parameters (S2 Table). While the factorial analysis showed significant effects of 2 factors on a dependent variable for all plant parameters at 125 DAT (S3 Table). The root fresh and dry weight was not significantly different for all bacterial strains. Therefore, the shoot and root weight were affected directly from water levels; single inoculum and co-inocula of the endophytic bacteria showed not significant differences in drought stress.

Drought stress strongly affected the yield of Jerusalem artichoke tubers, including fresh and dry weight, inulin content and harvest index (Table 2), but had no effect on the overall number of tubers. The single inoculation increased the tuber weight under well-watering compared with the control, giving 9% and 18% induction of fresh and dry tuber weight, respectively; in contrast, the co-inocula of *R*. *aquimaris* 3.13+*B*. *velezensis* 5.18 and triple-inoculation significantly increased the tuber weight with an average 52% and 90% increase of fresh and dry tuber weight, respectively. The single inoculation of *R*. *aquimaris* 3.13 showed a remarkable increase (100%) in total fresh and dry weights of tubers under drought stress. The co-inocula of *R*. *aquimaris* 3.13+ *B*. *velezensis* 5.18 induced 49% and 67% of the fresh and dry weight of tubers under drought conditions, respectively. The factorial analysis revealed that the water levels had no major impact on tuber weight; the bacterial strains individually affected tuber weight. The highest inulin content, a valuable product, was evident in the co-inocula of *R*. *aquimaris* 3.13+*B*. *velezensis* 5.18 under well-watering with a 10% increase from the control. The single inoculation failed to enhance inulin under well-watering. Inulin content was lower under drought stress, but the plants inoculated with the single and co-inocula of the endophytic bacteria increased in inulin accumulation at an average of 16% and 24%, respectively. *R*. *aquimaris* 3.13, *B*. *velezensis* 5.18 and the co-inocula of *R*. *aquimaris* 3.13+ *M*. *luteus* 4.43, *R*. *aquimaris* 3.13+*B*. *velezensis* 5.18 and triple-inocula promoted inulin content significantly under drought condition. Thus, the harvest index failed to exhibit significant differences with the control under drought condition, but it did show significant differences in well-watering for the single inoculation.

**Table 2:** Yield of Jerusalem artichoke under well-watering and drought condition at 125 days of harvest expressed as tuber fresh weight (TFW), tuber dry weight (TDW), tuber number (TN), inulin content (IN) and harvest index (HI).

Water level	Treatment	TFW (g plant ^-1^)	TDW (g plant ^-1^)	TN (plant^-1^)	IN (%)	HI
W1	T1	41.57±4.90^de^	11.01±0.62^cd^	6.25±1.25	70.08±4.60^b^	6.49±0.03^a-c^
	T2	52.99±7.21^b-d^	14.25±2.62^b-d^	5.00±0.00	72.34±2.76^ab^	6.14±0.04^b-d^
	T3	51.45±14.22^d^	13.82±3.80^cd^	4.75±0.96	69.40±3.47^b^	6.96±0.06^ab^
	T4	51.92±5.83^cd^	14.65±2.65^b-d^	4.75±1.50	72.18±7.01^ab^	5.87±0.05^c-e^
	T5	67.27±12.53^a-c^	22.02±7.43^a^	7.75±4.86	76.69±4.59^a^	5.27±0.07^d-f^
	T6	40.94±6.02^de^	10.46±1.57^cd^	6.00±4.80	73.36±4.05^ab^	5.87±0.03^c-e^
	T7	68.48±25.56^ab^	20.18±9.78^ab^	5.50±1.73	71.68±2.60^ab^	7.32±0.14^a^
	T8	43.51±6.85^de^	11.22±0.59^cd^	5.00±0.82	69.87±2.53^b^	5.09±0.02^ef^
W2	T1	73.71±11.58^a^	24.61±6.31^a^	6.25±2.63	52.55±6.63^c^	4.38±0.08^f^
	T2	49.75±9.00^de^	13.62±3.06^cd^	5.00±0.82	45.65±3.03^de^	5.29±0.05^d-f^
	T3	47.55±6.69^de^	13.14±2.43^cd^	4.25±1.71	51.01±2.44^cd^	5.06±0.06^ef^
	T4	48.18±11.77^de^	13.52±4.53^cd^	4.50±1.29	52.22±3.42^c^	5.06±0.08^ef^
	T5	52.01±4.36^cd^	15.02±1.69^bc^	4.50±0.58	52.94±4.13^c^	5.14±0.02^ef^
	T6	40.54±4.98^de^	10.47±1.12^cd^	5.00±2.00	47.14±2.66^c-e^	4.92±0.02^f^
	T7	35.75±10.80^e^	8.80±2.85^d^	7.00±0.82	52.25±4.84^c^	5.24±0.07^d-f^
	T8	35.80±9.85^e^	8.95±2.65^d^	5.00±2.16	41.41±0.61^e^	5.04±0.08^ef^
F for WxI		5.43*	5.77*	0.79	2.15	4.68*
%CV		21.88	29.76	39.37	6.75	14.56

Values with different superscripts are significantly different at P < 0.01 probability levels, except TN is no significant difference.

* is significantly different at P < 0.01 probability levels. W1 = 100% AW, W 2 = 1/3 AW. Treatment = T1: *R*. *aquimaris* 3.13, T2: *M*. *luteus* 4.43, T3: *B*. *velezensis* 5.18, T4: *R*. *aquimaris* 3.13+ *M*. *luteus* 4.43, T5: *R*. *aquimaris* 3.13+*B*. *velezensis* 5.18, T6: *M*. *luteus* 4.43+*B*. *velezensis* 5.18, T7: *R*. *aquimaris* 3.13+ *M*. *luteus* 4.43+*B*. *velezensis* 5.18 and T8: Control. W means water level and I means bacterial isolates.

### Roles of endophytic bacteria to root and leaf growth parameters

**Fig 3:**
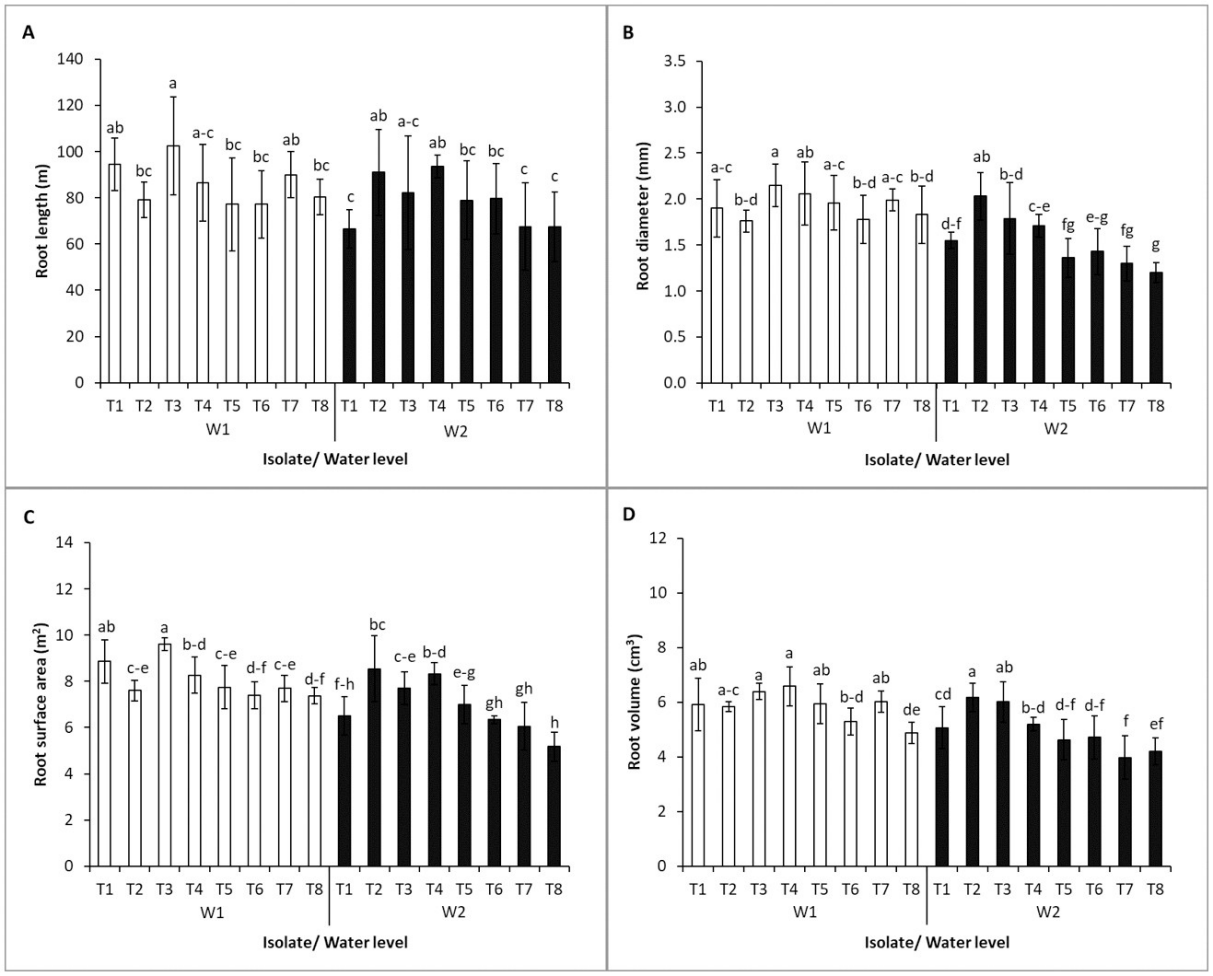
Root parameters (length, diameter, surface area, volume) of Jerusalem artichoke due to planting in normal (W1) and drought condition (W2) inoculated with single inoculum and co-inocula of endophytic bacteria at 125 days after planting (harvest time). T1-T8 represent treatment of planting described in Table 1. Bars mean standard deviation followed by the different letters that are significantly different at P<0.05.

Single inoculum or co-inocula of endophytic bacteria had no effect on the root length according to the factorial analysis with no significant differences at harvest time (S3 Table). Water levels strongly affected root traits in this study. Only *B*. *velezensis* 5.18 was outstanding for root length promotion significantly differing from the control under well-watering (Fig 3). In drought stress, *M*. *luteus* 4.43 and the co-inocula of *R*. *aquimaris* 3.13+ *M*. *luteus* 4.43 indicated major promotion in root length. *B*. *velezensis* 5.18 enhanced significantly the root diameter and root surface under well-watering; also, *R*. *aquimaris* 3.13 induced the root surface. The single inoculation showed significant effect on the root diameter under drought stress; the co-inocula of *R*. *aquimaris* 3.13+ *M*. *luteus* 4.43 also induced the root diameter. For the root surface in drought, *M*. *luteus* 4.43 and *B*. *velezensis* 5.18 showed significant differences from control as same as the co-inocula of *R*. *aquimaris* 3.13+ *M*. *luteus* 4.43 and *R*. *aquimaris* 3.13+*B*. *velezensis* 5.18 strains. The single inoculation clearly promoted root volume in both water levels. Co-inoculation promoted root volume under well-watering, except the co-inocula of *M*. *luteus* 4.43+*B*. *velezensis* 5.18; only the co-inocula of *R*. *aquimaris* 3.13+*M*. *luteus* 4.43 aided root volume increase under drought condition.

The factorial analysis at 125 days (harvest time) showed that the endophytic bacteria affected chlorophyll concentrations (SPAD), leaf area (LA), photosynthetic rate (Pn) and water use efficiency (WUE) (S3 Table). Moreover, LA and WUE of bacteria inoculated plants did not differ from the control under drought stress; triple-inoculation yielded the highest values of LA and WUE under well -watering condition (Table 3). The single inoculum of *M*. *luteus* 4.43 showed high LA and *R*. *aquimaris* 3.13 and *B*. *velezensis* 5.18 led to high WUE under well watering. The SPAD values were notably high in *B*. *velezensis* 5.18 and *R*. *aquimaris* 3.13+*B*. *velezensis* 5.18 under drought condition. Significantly, the single and the co-inoculation exhibited higher the Pn value than the control in both conditions; the co-inoculation was more effective than the single inoculation, increasing the Pn value more than 21%. The SPAD values were not related to Pn values, leading to individual effects from the bacterial strains.

**Table 3:** Plant parameters in different water levels. SPAD, Leaf area (LA), Photosynthetic rate (Pn), Water use efficiency (WUE), Stomatal conductance (Gs) and Transpiration rate (Tr) of Jerusalem artichoke at 125 days after transplanting (DAT) with endophytic bacteria in different water levels.

Water level	Treatment	SPAD	LA (m^2^plant^-1^)	Pn (μmol CO_2_ m^-2^ s^-1^)	WUE (μmol CO_2_ /mmol H_2_O)	Gs (H_2_O m^-2^ s^-1^)	Tr (mmol H_2_O m^-2^ s^-1^)
W1	T1	34.87±1.74^h^	7.10±1.62^d-f^	10.33±0.30^de^	6.49±0.57^a-c^	0.11±0.02^a-d^	1.89±0.14^cd^
	T2	36.70±1.82^d-h^	8.87±1.11^bc^	12.97±1.54^ab^	6.14±0.88^b-d^	0.15±0.06^a^	2.68±0.56^a^
	T3	40.10±1.50^a-c^	8.28±1.25^b-d^	11.60±1.05^a-d^	6.96±0.84^ab^	0.08±0.01^d^	1.71±0.17^d^
	T4	35.67±1.09^gh^	7.56±1.26^b-e^	12.24±0.87^bc^	5.83±0.51^c-e^	0.14±0.04^ab^	2.41±0.38^a-c^
	T5	38.60±1.66^a-d^	8.15±0.92^b-d^	13.62±1.24^a^	5.27±0.35^d-f^	0.11±0.02^a-d^	1.98±0.28^b-d^
	T6	36.05±2.45^e-h^	9.15±1.49^ab^	11.90±1.55^bc^	5.87±0.24^c-e^	0.11±0.02^a-d^	2.12±0.44^b-d^
	T7	38.37±0.55^b-e^	10.69±1.58^a^	11.37±1.03^cd^	7.32±1.07^a^	0.09±0.02^cd^	1.17±0.52^d^
	T8	34.72±1.22^h^	6.91±0.30^d-f^	9.64±1.15^ef^	5.09±0.72^ef^	0.14±0.03^ab^	2.21±0.22^a-d^
W2	T1	35.87±1.71^f-h^	6.99±1.21^d-f^	9.13±0.32^e-g^	4.38±0.83^f^	0.11±0.02^a-d^	2.15±0.42^a-d^
	T2	37.97±0.91^c-g^	6.70±0.91^d-f^	8.08±1.50^gh^	5.29±0.35^d-f^	0.08±0.02^d^	1.71±0.28^d^
	T3	40.65±2.15^ab^	7.65±0.15^b-e^	8.23±0.44^gh^	5.06±0.51^ef^	0.10±0.02^b-d^	2.12±0.53^b-d^
	T4	32.27±1.36^b-f^	6.40±1.07^ef^	8.71±1.04^fg^	5.07±0.60^ef^	0.08±0.01^d^	1.72±0.27^d^
	T5	41.02±0.63^a^	5.51±0.59^f^	8.50±0.74^fg^	5.14±0.50^ef^	0.12±0.02^a-c^	2.05±0.38^b-d^
	T6	35.10±1.07^d-h^	6.37±0.95^ef^	9.34±0.58^e-g^	4.92±0.87^f^	0.11±0.00^a-d^	2.02±0.24^b-d^
	T7	37.05±3.49^h^	7.86±1.34^b-e^	9.44±0.31^e-g^	5.24±0.38^d-f^	0.13±0.03^a-c^	2.44±0.61^ab^
	T8	38.00±0.53^c-g^	7.47±0.29^c-e^	7.00±0.74^h^	5.04±0.40^ef^	0.09±0.03^cd^	1.73±0.44^d^
F for WxI		2.72*	2.76*	3.93**	3.20**	3.58**	4.48**
%CV		4.56	14.76	9.63	11.78	27.19	19.01

Values with different superscripts are significantly different in each column at P < 0.01 probability levels, except LA is significantly different at P < 0.05 probability levels.

* and ** are significantly different at P < 0.05 and P < 0.01 probability levels, respectively. W1 = 100% AW, W 2 = 1/3 AW. Treatment = T1: *R*. *aquimaris* 3.13, T2: *M*. *luteus* 4.43, T3: *B*. *velezensis* 5.18, T4: *R*. *aquimaris* 3.13+*M*. *luteus* 4.43, T5: *R*. *aquimaris* 3.13+*B*. *velezensis* 5.18, T6: *M*. *luteus* 4.43+*B*. *velezensis* 5.18, T7: *R*. *aquimaris* 3.13+*M*. *luteus* 4.43+*B*. *velezensis* 5.18 and T8: Control. W means water level and I means bacterial isolates.

### Endophytic bacteria mediated changes in gene response

**Fig 4:**
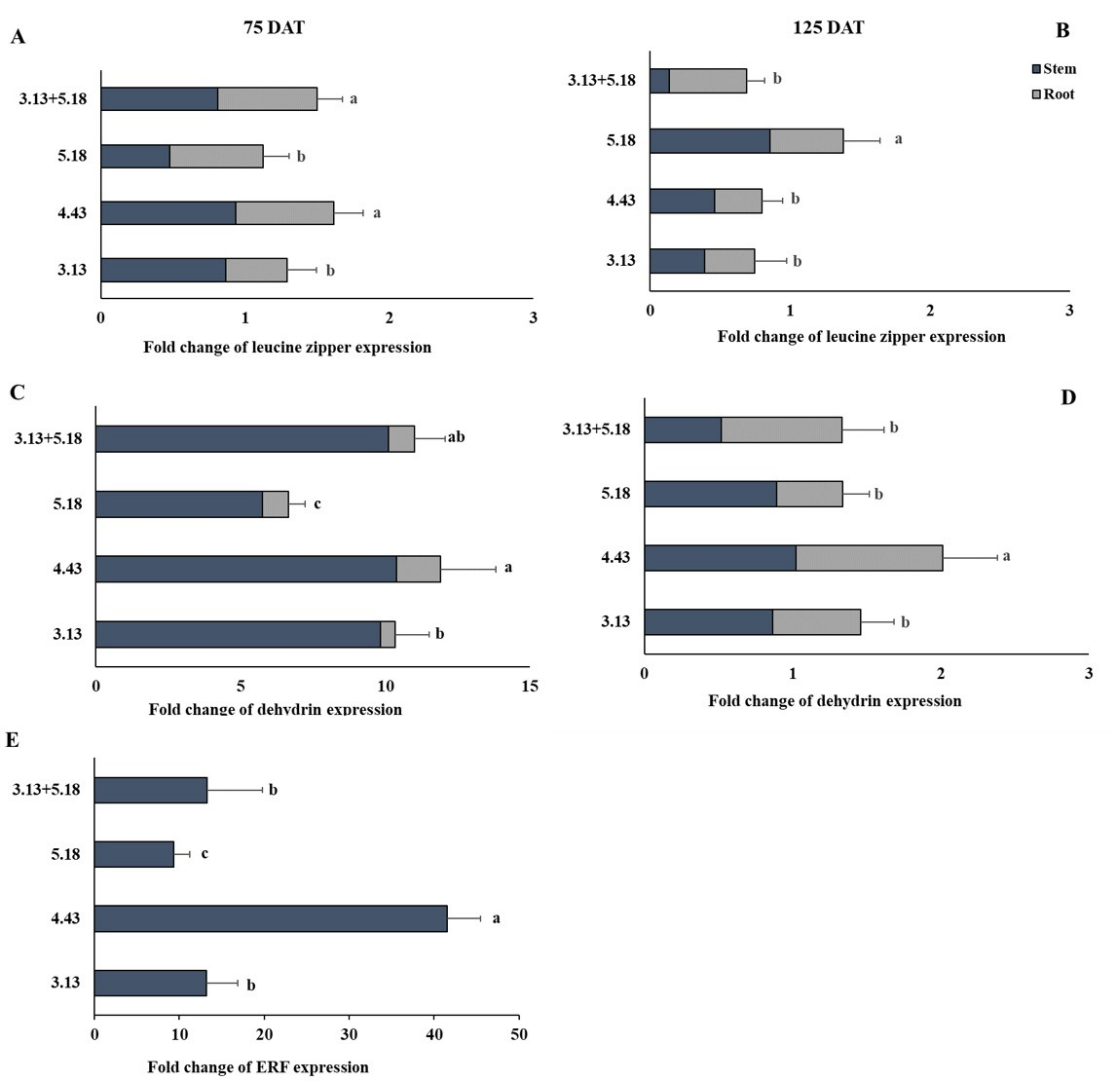
Gene expression of leucine zipper (A, B) dehydrin like protein (C, D) and ethylene responsive element binding factor 1 (ERF1) (E) in stem and root of Jerusalem artichoke at 75 and 125 days after inoculation with the endophytic bacteria (*R*. *aquimaris* 3.13, *M*. *luteus* 4.43, *B*. *velezensis* 5.18 and the mixed culture of *R*. *aquimaris* 3.13+*B*. *velezensis* 5.18) under drought condition. Bars mean standard deviation followed by the different letters that are significantly different at P<0.05.

**Fig 5:**
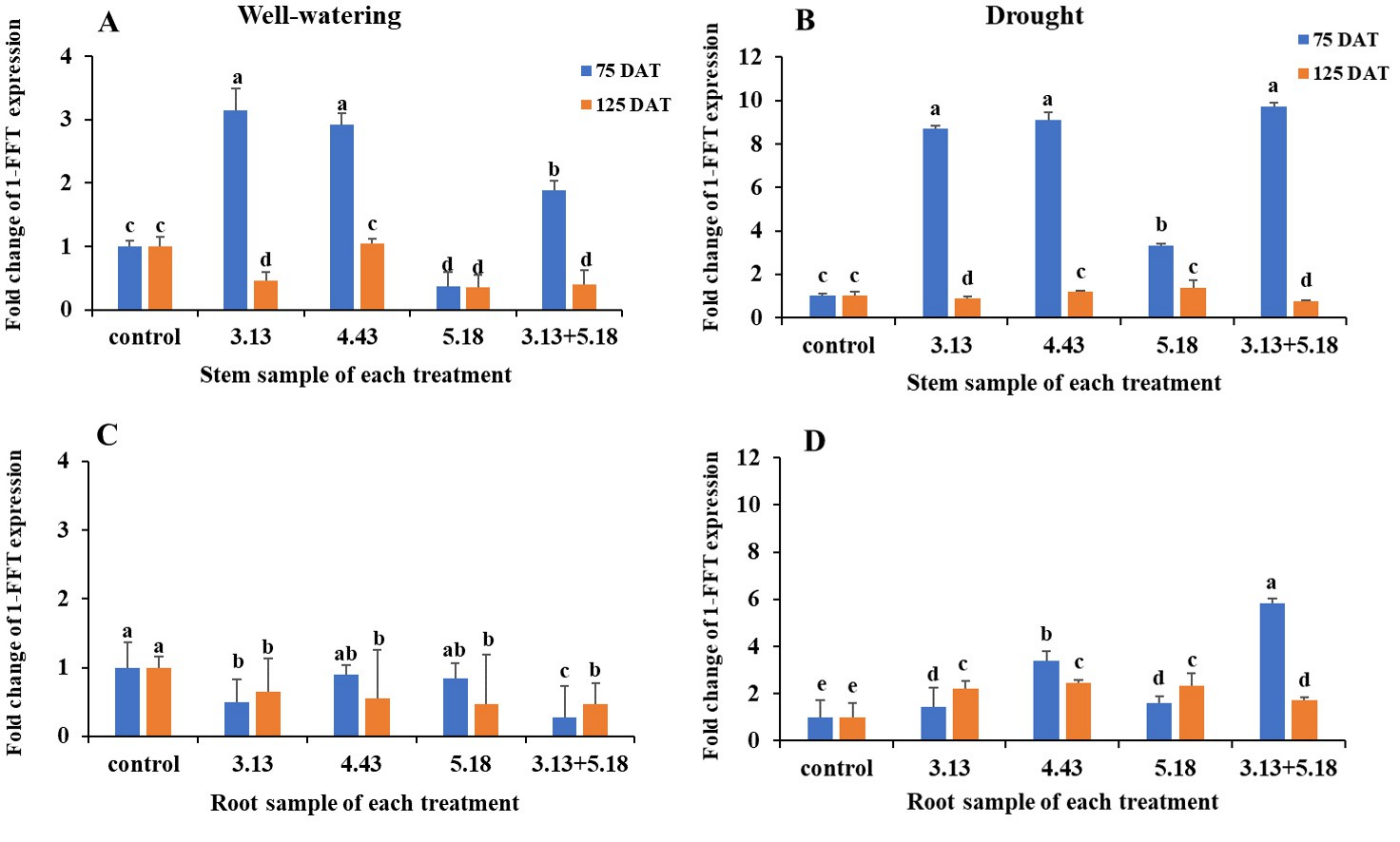
The 1-FFT gene expression in stem and root of Jerusalem artichoke at 75 and 125 days after inoculation with the endophytic bacteria (*R*. *aquimaris* 3.13, *M*. *luteus* 4.43, *B*. *velezensis* 5.18 and the mixed culture of *R*. *aquimaris* 3.13+*B*. *velezensis* 5.18) under well-watering and drought condition. Bars mean standard deviation followed by the different letters that are significantly different at P<0.05.

The homeobox-leucine zipper (HD-Zip) gene showed downregulation in all treatments at 75 DAT and 125 DAT (Fig 4) compared with the control expressing gene at value 2 in total of stem and root. Moreover, the expression of dehydrin gene showed considerable upregulation in all bacterial treatments at 75 DAT, particularly *M*. *luteus* 4.43 (with more than 5-10-fold expression). The dehydrin gene of plants inoculated with the 5.18 strain expressed the lowest value compared with other strains. The ethylene responsive element binding factor 1 (ERF1) gene exhibited outstanding up-regulated expression only in the stems inoculated with all endophytic bacteria, particularly different in *M*. *luteus* 4.43 (40-fold expression). No ERF1 gene expression was detected at harvest time. The results indicated that the endophytic bacteria involved in the expression of dehydrin and ERF1 gene responded to drought stress. Other genes involved in inulin synthesis of Jerusalem artichoke, 1-SST and 1-FEH, could not be detected in both well-watered and drought conditions; meanwhile 1-FFT gene was observed in both stem and root during planting until harvest time, and it showed constant expression in stem and root of the control treatments under well-watering and drought until harvest time (Fig 5). Expression of 1-FFT gene was up-regulated in the stem of the plants inoculated with *R*. *aquimaris* 3.13 and *M*. *luteus* 4.43, subsequently the co-inocula of *R*. *aquimaris* 3.13+*B*. *velezensis* 5.18 at 75 DAT under well-watering; the downregulation of this gene showed in both stem and root at 125 DAT. This gene was significant upregulation under drought condition due to the endophytic bacteria in both stem and root at early stage of harvest. The outstanding strains were *M*. *luteus* 4.43, *R*. *aquimaris* 3.13 and the co-inocula of *R*. *aquimaris* 3.13+*B*. *velezensis* 5.18 according to inulin yield.

**Fig 6:**
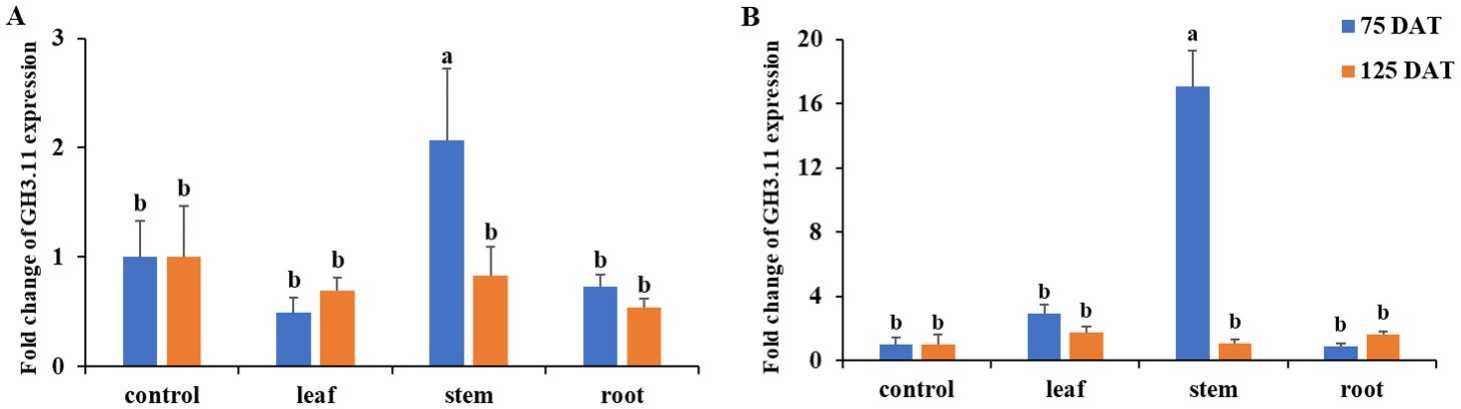
The GH3.11 gene expression in stem, leaf and root of Jerusalem artichoke at 75 and 125 days after inoculation with *M*. *luteus* 4.43 under well-watering (A) and drought condition (B). Bars mean standard deviation followed by the different letters that are significantly different at P<0.05.

Only plants inoculated with *M*. *luteus* 4.43 expressed the gene IAA amido synthetase (GH3.11), in both well-watering and drought conditions. The GH3.11 gene was significant up-regulated in the stem of Jerusalem artichoke at 75 DAT expressing at 2-fold and 17-fold higher than the control in well-watering and drought, respectively (Fig 6).

## Discussion

Endophytic bacteria live in plant tissues and play important roles promoting plant growth in normal and stress conditions through the production of IAA auxin, ACC deaminase, exopolysaccharide and some osmolytes. In this study, each strain of endophytic bacteria exhibited its abilities to promote plant growth under drought condition, while some strains possessed genes involved in inulin production. *Micrococcus luteus* 4.43 enabled IAA production in normal condition and under water limitation [19]; meanwhile *Rossellomorea aquimaris* 3.13 degraded inulin (inulinase), yielding fructose, fructooligosaccharide and *Bacillus velezensis* 5.18 promoted inulin synthesis (levansucrase), giving inulin or levan protecting osmotic pressure [21]. Co-inoculation of these bacteria might be an effective combination to assist plant growth promotion under well-watering condition whilst also aiding plant response in drought stress; thus, single inoculum and co-inocula of these bacteria were studied. Increased height of Jerusalem artichoke observed in the bacterial inoculation might involve the function of IAA (induction of cell division and elongation) obtaining *R*. *aquimaris* 3.13 and *M*. *luteus* 4.43 and ACC deaminase (lower ethylene production) acquiring *R*. *aquimaris* 3.13 and *B*. *velezensis* 5.18 [29]. Certainly, the single inoculum of *M*. *luteus* 4.43 with strong IAA production promoted plant height and had synergistic action with *R*. *aquimaris* 3.13 and *B*. *velezensis* 5.18 under well-watering. *B*. *velezensis* 5.18 increased plant height under drought; this could mean that IAA has a direct function in different physiological processes, leading to plant growth promotion. Besides IAA, ACC deaminase, obtained in *B*. *velezensis* 5.18, decreases ethylene production in plants and ultimately increases plant growth. Moreover, *B*. *velezensis* 5.18 produced levan (fructooligosaccharides) which may adjust osmotic potential inside the plant cells and be utilized as a nutrient source for plant growth. The single inoculation of *M*. *luteus* 4.43 and *B*. *velezensis* 5.18 resulted in more benefits than the co-inoculation in root traits under well-watering and drought stress. The co-inocula of *R*. *aquimaris* 3.13+*B*. *velezensis* 5.18 was a negative combination for plant growth; this might be due to their functions in inulin synthesis and accumulation, resulting in high inulin yield. A possible reason is the induction of 1-FFT gene expression in the stem by *R*. *aquimaris* 3.13 at 75 and 125 DAT under well-watering. The inulinase of *R*. *aquimaris* 3.13 may digest oligosaccharides in plant stems after which plants use trisaccharide to make fructan chains by the function of 1-FFT, leading to fructan levels in relation to inulin accumulation in tubers of Jerusalem artichoke. This indicates that *R*. *aquimaris* 3.13 obtained the inulinase involved in inulin synthesis via 1-FFT which is one of the enzymes in the pathway of fructan synthesis in plants, occurring by elongation of trisaccharide (1-kestose, 6-kestose, neokestose) to produce inulin, neolevan, and graminan [30]. *B*. *velezensis* 5.18 strongly promoted root development (length, diameter, surface, volume), leading to high biomass (root and shoot weight). It is possible that this strain possessed levansucrase [21] and produced fructooligosaccharide to prolong fructans which are involved in osmotic adjustment for plant growth and tolerance [31]. The osmoprotectants are sugars (e.g., sucrose), glycine betaine, organic acids (e.g., malate), inorganic ions (e.g., calcium), and proline which protect proteins, organelles, membranes, and genetic materials against stress, including balance of osmotic pressure [32]. It is likely that fructans accumulated in plants against abiotic stresses may contribute to overall cellular reactive oxygen species (ROS) homeostasis by direct ROS scavenging mechanisms; small fructans may act as phloem-mobile signaling compounds under stress [33].

The evidence clearly demonstrates that under drought condition inoculation with endophytic bacteria induced inulin accumulation, except with *M*. *luteus* 4.43 and co-inoculation of *M*. *luteus* 4.43+*B*. *velezensis* 5.18. According to the induction of 1-FFT gene expression observed in stem and root at 75 and 125 DAT of all treatments under drought. *R*. *aquimaris* 3.13 carrying inulinase digested oligosaccharides; another strain (*B*. *velezensis* 5.18) produced levansucrase for polysaccharides production. Thus, sugar products could be nutrients, osmolytes or precursors of 1-FFT. However, inulin synthesis within Jerusalem artichoke required 1-SST and 1-FFT to produce short chain inulin in tubers and FEHs which are inulinase, a degradation of inulin. Normally, 1-SST exhibits high expression at an early stage of plant growth and decreases along growing time until harvest, while the expression of 1-FFT remains constant [34]. The reduction of 1-SST transcription and a parallel increase of the transcription of FEHs genes resulted in a reduction of inulin chain length and an increase of free sugars. To produce long chain inulin, the overexpression of 1-SST is an important criterion. In this work, 1-SST and 1-FEH genes were not observed in both well-watering and drought conditions; perhaps the expression of 1-SST was very low at late growing stages and 1-FEH may be suppressed by drought in order to reserve inulin as a food source. Thus, the endophytic bacteria carrying inulinase and levansucrase could cope with the loss of 1-SST and 1-FEH functions under drought. Moreover, *R*. *aquimaris* 3.13 and *B*. *velezensis* 5.18 were involved in high Pn and SPAD, respectively, whilst the co-inocula of *R*. *aquimaris* 3.13+*B*. *velezensis* 5.18 also induced SPAD of plant under drought. This evidence reveals that endophytic bacteria may be involved in photosynthesis and the chlorophyll content of the plant host. Thus, *R*. *aquimaris* 3.13 and *B*. *velezensis* 5.18 strains should be candidates for application in other fructan-producing plants, such as potato, by the utilization of either single inoculum or co-inocula.

The dehydrin like protein is one of the stress response genes involved in water stimulus and chemical stimulus observed in both the leaves and root of Sunflower (*Helianthus annuus*) after 24 hours of drought stress (15% PEG 6000) [35]. This gene was detected in stems of Jerusalem artichoke at high expression under drought for all treatments inoculated with the endophytic bacteria at 75 DAT. Dehydrins accumulate during late embryogenesis or in young plant organs for cell division or cell elongation, e.g., root tips and elongating stems [9]. The expression of dehydrin is strongly stimulated by abiotic stress through multiple signaling pathways, e.g., abscisic acid (ABA) or phytohormones [36]. Results from present study implied that endophytic bacteria helped plant response to drought by the stimulation of the dehydrin like protein. The ethylene responsive element binding factor 1 (ERF1) is an early ethylene responsive gene related to abiotic stress (salinity and drought stress). The ERF1 gene is activated by salt, drought, and heat with stress-specific gene regulation, which integrates jasmonate, ethylene, and abscisic acid signals. Over-expression of ERF1 could enhance abiotic stress tolerance in many plants, e.g., *Arabidopsis* [37], wheat [38], tobacco [39]. In Jerusalem artichoke, ERF1 exhibited very high expression under early drought (75 DAT) in all treatments inoculated with endophytic bacteria. *M*. *luteus* 4.43 enhanced ERF1 expression 40-fold compared with the control; this strain probably activated the ERF1 via ethylene signal from IAA stimulation. Another drought gene response, the HD-Zip is an important gene family involved in plant growth and development and has potential in enhancing plant tolerance to various abiotic and biotic stresses, particularly in HD-Zip I subfamily genes that responded to drought and salinity stress [40]. However, in this study the HD-Zip gene was stimulated by endophytic bacteria in low expression; this gene might display a strong response and stimulation from drought stress.

IAA amido synthetase (GH3.11) is a gene response to stress involved in hormone stimulus, endogenous stimulus, organic substance, chemical stimulus and radiation [35]. This gene was investigated in the plants inoculated with *M*. *luteus* 4.43 and the results showed high expression in drought stress at 75 DAT. Moreover, the effects of *M*. *luteus* 4.43 on plant growth were indicated in all root parameters (length, diameter, surface and volume) under drought condition. The results support IAA producing bacteria involvement in root proliferation, leading to greater nutrient uptake, due to *M*. *luteus* 4.43 induction of GH3.11, the dehydrin like protein and ERF1 gene expression under drought. This means that IAA was a signal involved in plant response for IAA synthesis and drought stress. Interestingly, this strain showed high induction of 1-FFT gene expression under well-watering and drought condition but no evidence of yield promotion (tuber weight and inulin) was demonstrated. This could mean that *M*. *luteus* 4.43 induced the IAA system, which is involved in many genes’ response to abiotic stress; the activation of various genes may be regulated by different components or networks.

## Conclusion

All endophytic bacteria demonstrated great potential for plant growth promotion, including yield, of Jerusalem artichoke. Comparison between single inoculum and co-inocula of the endophytic bacteria concluded that single inoculum of the endophytic bacteria promoted plant growth well (shoot and root weight, root length, diameter, surface and volumes), while co-inocula of the endophytic bacteria had good promotion in yield (tuber weight, inulin and harvest index). This applied to both well-watering and drought conditions. The endophytic bacteria were involved in the stimulation of plant genes, dehydrin like protein and ERF1, for adaptation to and survival in drought conditions; they were also involved in the stimulation of 1-FFT gene for the production of inulin and protectant molecules (nutrient or osmolytes). This work demonstrated the functions and interactions of the endophytic bacteria with a plant host. The potential strains were *M*. *luteus* 4.43 and *B*. *velezensis* 5.18 for plant growth promotion by single inoculation in both well-watering and drought conditions; in addition, the co-inoculation of *R*. *aquimaris* 3.13+*B*. *velezensis* 5.18 was an inoculant for inulin production which may be involved in plants accumulating inulin, sugar or starch. The formulation of a single strain should be studied for application using single inoculation or combination. As the intensity and nature of the benefit of microbial inoculation vary with soil type and plant growth conditions, further studies involving different soil types and growing conditions are essential to reveal the true potential of these bacterial isolates.

## Supporting information

S1 Table Primer of quantitative real time PCR used to evaluate plant gene responses. (DOC)

S2 Table Factorial analysis of plant growth. Factorial analysis of endophytic bacteria and different water levels at 75 days after transplanting.
(DOC)

S3 Table Factorial analysis of plant growth. Factorial analysis of endophytic bacteria and different water levels at 125 days after transplanting.
(DOC)
