# Supplementary material for: Single and co-inoculum of endophytic bacteria promote growth and yield of Jerusalem artichoke through upregulation of plant genes under drought stress
Source: PLoS One. 2023 Jun 2;18(6):e0286625. doi: 10.1371/journal.pone.0286625 (PMC10237377; doi:10.1371/journal.pone.0286625)
Supplement: S1 Table — (DOC) [file pone.0286625.s001.doc]

S1 Table. Primer of quantitative real time PCR used to evaluate plant gene responses

| Primer | Gene | Sequence (5’-3’) | Reference |
| --- | --- | --- | --- |
| LeuZip-F | Homeobox-leucine zipper protein | GCTTGCGTCTAAATCCGAGTC | [14] |
| LeuZip-R |  | ATTCTTCACCGCTGCCACTAC |  |
| Dehydrate-F | dehydrin like protein | GGCATTCTGCATCGTTCTGG | [14] |
| Dehydrate-R |  | CTGATACTGCTCCTCTTGTCTGTGA |  |
| ERF1A-F | ethylene responsive element binding factor 1 | TCCTCAACGACGCCTTTCAC | [15] |
| ERF1A-R |  | CACTCCTCTATAATGCTTTCCCT |  |
| 1_SST_F | sucrose:sucrose 1-fructosyltransferases | TACCATTTTCAACCCGATAAGAAT | [16] |
| 1_SST_R |  | CATGCTCTACACTGGCAACG |  |
| 1_FFT_F | fructan:fructan 1-fructosyltransferase | TGCGATTACGGAAGGTTCTT | [17] |
| 1_FFT_R |  | CAACATTATAGATTGTAGCCCATCC |  |
| 1_FEH_F | fructan β-(2,1)-fructosidase/1-exohydrolase | GGCGGATGTTACAATTTCGT | [17] |
| 1_FEH_R |  | AAACCAACTTGGGCGATA |  |
| GH3.11-F | IAA amido synthetase | CACAATTTCGGTTCATCT | [18] |
| GH3.11-R |  | TCAAGCACTTTAGGGACA |  |
| Actin-F | Actin | GGTATTCACGAGACCACCTACAACT | [14] |
| Actin-R |  | CCTCCGATCCAGACACTGTATT |  |
