# Supplementary material for: Single and co-inoculum of endophytic bacteria promote growth and yield of Jerusalem artichoke through upregulation of plant genes under drought stress
Source: PLoS One. 2023 Jun 2;18(6):e0286625. doi: 10.1371/journal.pone.0286625 (PMC10237377; doi:10.1371/journal.pone.0286625)
Supplement: S2 Table — Factorial analysis of endophytic bacteria and different water levels at 75 days after transplanting. (DOC) [file pone.0286625.s002.doc]

**S2 Table.** Factorial analysis of endophytic bacteria and different water levels at 75 days after transplanting

| SOV | Df | SPAD | Height | LA | FWS | FWR | DWS | DWR | Pn | WUE |
| --- | --- | --- | --- | --- | --- | --- | --- | --- | --- | --- |
| REP | 3 | 0.83 | 4.00 | 0.10E+05 | 32.79 | 1.20 | 0.57 | 0.02 | 0.23 | 0.99 |
| W | 1 | 112.89** | 862.89** | 1.63E+05** | 540.15** | 170.59** | 93.79** | 7.41** | 313.73** | 28.83** |
| I | 7 | 22.62** | 123.43** | 1.35E+05** | 87.81** | 16.25** | 6.38** | 0.40** | 9.18** | 0.99** |
| WxI | 7 | 16.47** | 50.24** | 0.34E+05** | 36.31** | 18.82** | 2.17** | 0.16* | 1.53* | 1.27** |
| F for WxI |  | 3.03 | 5.42 | 6.25 | 2.73 | 3.53 | 3.27 | 2.46 | 2.21 | 4.12 |
| %CV |  | 6.62 | 5.80 | 7.82 | 10.84 | 16.83 | 10.05 | 19.15 | 4.01 | 7.57 |

LA: Leaf area; FWS: Fresh weight of shoot; FWR: Fresh weight of root; DWS: Dry weight of shoot; DWR: Dry weight of root; Pn: Photosynthetic rate; WUE: Water use efficiency.

| SOV | Df | Gs | Tr | SMC | RL | RD | RV | RS | RWC |
| --- | --- | --- | --- | --- | --- | --- | --- | --- | --- |
| REP | 3 | 0.00 | 0.08 | 1.40 | 0.72E+07 | 0.04 | 5.26 | 0.77E+05 | 6.06 |
| W | 1 | 0.00** | 3.11** | 586.54** | 4.05E+07** | 3.08** | 26.51** | 4.10E+05** | 6642.66** |
| I | 7 | 0.00** | 0.20* | 1.04ns | 0.60E+07* | 0.31** | 3.91** | 0.59E+05** | 126.59** |
| WxI | 7 | 0.00** | 0.32* | 1.71* | 0.63E+07* | 0.17** | 3.91** | 0.59E+05** | 28.59** |
| F for WxI |  | 6.07 | 3.11 | 2.52 | 2.35 | 4.41 | 2.88 | 2.58 | 3.86 |
| %CV |  | 9.99 | 10.24 | 7.60 | 22.11 | 12.03 | 22.01 | 21.54 | 3.47 |

Gs: Stomatal conductance; Tr: Transpiration rate; SMC: Soil Moisture Content; RL: Root length; RD: Diameter of root; RV: Root volume; RS: Root surface area; RWC: Relative water content.

** = P ≤ 0.01 probability levels, * = P ≤ 0.05 probability levels, ns = non-significant respectively.
