# Supplementary material for: Single and co-inoculum of endophytic bacteria promote growth and yield of Jerusalem artichoke through upregulation of plant genes under drought stress
Source: PLoS One. 2023 Jun 2;18(6):e0286625. doi: 10.1371/journal.pone.0286625 (PMC10237377; doi:10.1371/journal.pone.0286625)
Supplement: S3 Table — Factorial analysis of endophytic bacteria and different water levels at 125 days after transplanting. (DOC) [file pone.0286625.s003.doc]

**S3 Table.** Factorial analysis of endophytic bacteria and different water levels at 125 days after transplanting

| SOV | Df | SPAD | LA | Pn | WUE | Gs | Tr |
| --- | --- | --- | --- | --- | --- | --- | --- |
| REP | 3 | 1.04 | 0.05E+05 | 1.66 | 0.23 | 0.00 | 0.19 |
| W | 1 | 19.80** | 3.47E+05** | 159.83** | 19.68** | 0.00ns | 0.13ns |
| I | 7 | 24.16** | 0.50E+05** | 5.67** | 1.33** | 0.00ns | 0.05ns |
| WxI | 7 | 7.94** | 0.34E+05** | 3.74** | 1.37** | 0.00** | 00.67** |
| F for WxI |  | 2.72 | 2.76 | 3.93 | 3.20 | 3.58 | 4.48 |
| %CV |  | 4.56 | 14.76 | 9.63 | 11.78 | 27.19 | 19.01 |

LA: Leaf area; Pn: Photosynthetic rate; WUE: Water use efficiency.; Gs: Stomatal conductance; Tr: Transpiration rate

| SOV | Df | Height | FWS | FWR | DWS | DWR |
| --- | --- | --- | --- | --- | --- | --- |
| REP | 3 | 42.54 | 40.08 | 15.35 | 0.87 | 0.17 |
| W | 1 | 540.56** | 1787.81** | 86.21** | 207.93** | 7.43** |
| I | 7 | 197.78** | 134.30** | 8.89ns | 8.68** | 0.16ns |
| WxI | 7 | 132.70** | 37.83* | 10.72* | 2.32** | 0.23* |
| F for WxI |  | 9.04 | 2.41 | 2.16 | 2.71 | 2.43 |
| %CV |  | 4.91 | 11.74 | 13.67 | 7.46 | 13.21 |

FWS: Fresh weight of shoot; FWR: Fresh weight of root; DWS: Dry weight of shoot; DWR: Dry weight of root.

| SOV | Df | RL | RD | RV | RS | Tuber/plant | FWT | DWT | Inulin |
| --- | --- | --- | --- | --- | --- | --- | --- | --- | --- |
| REP | 3 | 7.56E+07 | 0.25 | 1.23 | 0.17E+05 | 2.56 | 60.43 | 12.95 | 8.38 |
| W | 1 | 9.09E+06* | 2.34** | 11.86** | 1.75E+05** | 3.06ns | 303.58ns | 22.89ns | 8137.84** |
| I | 7 | 3.26E+06ns | 0.20** | 2.63** | 0.46E+05** | 4.24ns | 400.65** | 72.55** | 57.30** |
| WxI | 7 | 4.57E+06* | 0.17** | 1.02** | 0.24E+05** | 3.59ns | 652.74** | 101.92** | 35.98** |
| F for WxI |  | 2.22 | 3.70 | 3.11 | 4.93 | 0.79 | 5.43 | 5.77 | 2.15 |
| %CV |  | 17.46 | 12.61 | 10.59 | 9.29 | 39.37 | 21.88 | 29.76 | 6.75 |

RL: Root length; RD: Diameter of root; RV: Root volume; RS: Root surface area; FWT: Fresh weight of tuber; DWT: Dry weight of tuber.

** = P ≤ 0.01 probability levels, * = P ≤ 0.05 probability levels, ns = non-significant respectively.
